# Frailty and Survivability of Polish Caucasian Nonagenarians and Centenarians

**DOI:** 10.3390/geriatrics9010014

**Published:** 2024-01-18

**Authors:** Agnieszka Skubiszewska, Katarzyna Broczek, Iwona Maruniak-Chudek, Gabriela Oledzka, Marta Izabela Jonas, Monika Puzianowska-Kuznicka, Malgorzata Mossakowska

**Affiliations:** 1 Department of Medical Biology, Medical University of Warsaw, 00-575 Warsaw, Poland; 2 Study on Ageing and Longevity, International Institute of Molecular and Cell Biology, 02-109 Warsaw, Poland; 3 Mazovia Branch, Polish Society of Gerontology, 01-826 Warsaw, Poland; 4 Faculty of Medical Sciences, Medical University of Silesia, 40-055 Katowice, Poland; 5 Department of Human Epigenetics, Mossakowski Medical Research Institute, Polish Academy of Sciences, 02-106 Warsaw, Poland; 6 Department of Geriatrics and Gerontology, Medical Centre of Postgraduate Education, 01-826 Warsaw, Poland

**Keywords:** frailty, longevity, physical functional performance, muscle strength dynamometer, mortality

## Abstract

Frailty is a major geriatric problem leading to an increased risk of disability and death. Prevention, identification, and treatment of frailty are important challenges in gerontology and public health. The study aimed to estimate the prevalence of the frailty phenotype (FP) among the oldest-old Polish Caucasians and investigate the relationship between the FP and mortality. Baseline data were collected from 289 long-lived individuals, including 87 centenarians and 202 subjects aged 94–99. Mortality was obtained from population registers over the following 5 years. Sixty percent of subjects were classified as frail, 33% as prefrail, and 7% as robust. Frailty was more common in women than men and among centenarians than nonagenarians. During the 5-year observation period, 92.6% of the frail women and all frail men died, while mortality rates were lower among prefrail, 78.8% and 66.7%, and robust individuals, 60% and 54.5%, respectively. In the survival analysis, frailty was the strongest negative risk factor: HR = 0.328 (95% CI: 0.200–0.539). The inability to perform handgrip strength measurement was an additional predictor of short survival. In conclusion, the FP is prevalent in nonagenarians and centenarians and correlates with lower survivability. Future studies should address differences between unavoidable age-associated frailty and reversible disability in long-lived individuals.

## 1. Introduction

Aging is a universal but highly heterogeneous process. Age-associated changes in physical and cognitive performance as well as functional status follow different trajectories dependent on numerous intrinsic, environmental, and epigenetic factors. Individual profiles of chronic diseases and their complications superimposed on physiological aging result in a variety of presentations, ranging from independency to severe and multidimensional disability. The heterogeneity of aging is especially visible in long-lived individuals. Research in this area is of major importance, as it might bring new insights into successful aging and its determinants. Interrelationships between aging, age-related diseases, and geriatric syndromes form the basis of geroscience as an interdisciplinary field of research [1,2].

The focus on frailty as one of the major geriatric problems has significantly increased over the last few decades. Frailty is defined as a reduction in the homeostatic reserve and increased susceptibility to stressors, leading to the augmented risk of disability or death [3]. Although frailty is strongly correlated with age, not all older individuals are affected, and, if diagnosed early, frailty might be reversible or attenuated by exercise, protein-calorie, and vitamin D supplementation or reduction of polypharmacy [3]. It is, however, clear that in most cases frailty leads to adverse health outcomes. In older adults, it is a stronger predictor of mortality risk than metabolic syndrome [4]. Females are affected with frailty more often than men but have a longer lifespan and this frailty–mortality paradox indicates the complexity of gender-related factors [5,6]. Multiple correlates of physical frailty were identified on biological, sociodemographic, and environmental levels, but age-related changes in muscle mass and function are considered the main mechanisms underlying the development of frailty [7,8,9]. Therefore, prevention, identification, and treatment of sarcopenia and frailty are important issues in gerontology, geriatrics, and public health [10]. Novel studies indicate that there are several groups of serum biomarkers related to the risk of frailty, including inflammation-related and muscle-related compounds [11].

Besides physical frailty, cognitive frailty has gained increased attention among researchers in the last decade. Brain aging leads to increased vulnerability due to a shrinking neurophysiological reserve. A consensus definition was developed in 2013 describing cognitive frailty as the simultaneous presence of physical frailty and mild cognitive impairment without dementia related to Alzheimer’s disease or other types of brain pathology [12]. Cognitive and physical frailty seem to be interrelated in a multidimensional way, and understanding common pathways of frailty might lead to practice-oriented solutions preventing adverse health outcomes [13,14].

Even though the theoretical construct of frailty is appealing and well-documented, screening for frailty is not routinely performed in daily clinical practice probably because it is not a specific disease but a geriatric syndrome characterized by a variety of signs and symptoms. Nevertheless, multiple frailty assessment instruments have been described and validated [15,16], and international guidelines for active screening for frailty and sarcopenia in older adults have been developed [3,10]. There has been a rising interest in frailty assessment for older adults requiring orthopedic surgery for fractures, as it may enhance evidence-based medicine and support the decision-making process, especially in the growing population of patients in advanced older age [17].

One of the commonly used scales is the frailty phenotype (FP) described by Fried et al. using five criteria: weight loss, slow walking speed, low level of physical activity, muscle weakness, and fatigue or feeling of exhaustion [18]. Individuals with three or more indicators are classified as frail, and those with one or two as prefrail. Muscle strength may be measured directly with the use of a dynamometer or assessed indirectly by a subjective evaluation of muscle function, e.g., the ability to lift objects [19]. The most popular and easy-to-administer measurement of muscle strength is handgrip strength (HGS).

The prevalence of frailty among people in advanced older age based on the FP was investigated in recent years. A study including five centenarian cohorts from Japan, Denmark, Sweden, France, and Switzerland indicated that its prevalence reached over 60% [20].

The present study aimed to estimate the prevalence of frailty among participants of the Polish Reference Genome for Genomic Diagnostics and Personalized Medicine research project (PlGen) within a subproject “Expanding the database of long-lived individuals for research on the genetics and epigenetics of aging. Multi-scale study” and to identify differences across sexes and individuals aged 94–99 years versus centenarians. Additionally, the relationship between the FP and mortality was investigated to define predictors of survivability in long-lived individuals. The main working hypotheses included the following: at least half of all respondents’ present symptoms of frailty, being nonfrail supports survivability, and handgrip strength measurement is a feasible and reliable method of muscle function assessment in advanced age in a Caucasian population.

## 2. Material and Methods 

### 2.1. Study Sample and Procedures 

**Figure 1:**
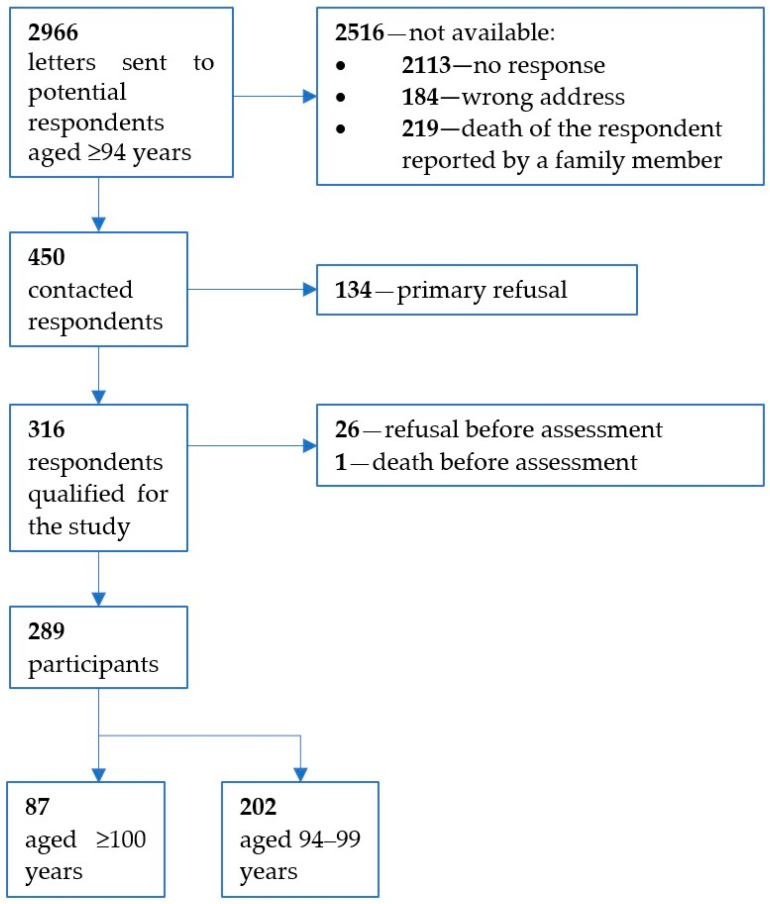
Study flow.

The study sample was recruited within the frame of the PlGen project dedicated to selecting long-lived, healthy individuals for whole genome sequencing to obtain a reference genome for genomic diagnostics and personalized medicine. The current study was a subproject of PLGen project, entitled “Expanding the database of long-lived individuals for research on the genetics and epigenetics of aging. Multi-scale study”. The expected sample size was 300 individuals aged 90 years or older. Invitations to participate in the study were sent by mail to 2966 citizens of Warsaw and its suburbs, who were identified in the Polish General Electronic System of Population Register (PESEL). Letters were sent successively, starting with the oldest people. Additionally, an attempt was made to contact all centenarians personally or by phone. The eligibility criterion was an informed consent form signed by the participant or a caregiver, including a consent to future use of data for research and publication purposes on the condition of confidentiality of individual data. The study flow is presented in Figure 1.

Face-to-face personal interviews were carried out between May 2013 and February 2015 by a single trained nurse. A questionnaire, originally used by The Five Country Oldest Old Project (5-COOP) research group [20], was translated into Polish. Baseline data were collected from 87 centenarians (67 women and 20 men, F:M ratio 3.4:1) and 202 individuals aged 94–99 years (158 women and 44 men, F:M ratio 3.6:1). 

In 42 cases, data were obtained from the participants only, in 33 cases mostly from the participants with a little assistance from their caregivers, in 202 both from the participants and the caregivers, and in 12 cases from the caregivers only. Elements of the comprehensive geriatric assessment (CGA) were performed directly after completing the questionnaire. 

All-cause five-year mortality was obtained using data from the PESEL database and city register offices. 

The project “Expanding the database of long-lived individuals for research on the genetics and epigenetics of aging. Multi-scale study” was approved by the Bioethics Committee of the Medical University of Warsaw (KB80/2012). The Bioethics Committee approved the following documents: an informed consent template, information for participants, and the research questionnaire. The study was conducted according to the guidelines of the Declaration of Helsinki.

### 2.2. Assessment of Frailty 

Frailty assessment was based on the frailty phenotype defined originally by Fried [18] and followed the 5-COOP study procedures [21]. Due to difficulties in obtaining reliable measurements of muscle strength and walking speed, the 5-COOP consortium decided to use the respondent’s self-assessment of weakness and slow gait [21]. The three remaining frailty criteria, unintentional weight loss, fatigue, and low level of physical activity, were defined according to Fried [18]. 

In the present study, the descriptive five-dimension FP included the following self-reported symptoms: (1) weight loss; (2) low outdoor activity and/or difficulty walking up stairs and/or difficulty to walk without rest; (3) slow or very slow walking speed or inability to walk; (4) difficulty carrying a 5 kg bag; (5) fatigue when moving or resting. Specific criteria for the above frailty symptoms are presented in Table 1. Participants were classified as robust if they met none of the listed criteria, as prefrail if they fulfilled 1 or 2 of them, and as frail if 3 or more symptoms were present. 

Additionally, an attempt to measure the handgrip strength (HGS) was undertaken in all subjects using a mechanical dynamometer (Baseline^®^ Smedley Spring Dynamometer, Fabrication Enterprises, White Plains, NY, USA). Participants were asked to hold the device at a right angle to their body in a sitting position and compress it with maximum force. In subjects who could not sit in a chair, HGS was measured in bed. The measurement was performed twice for each hand. The nurse performing measurements noted individual reasons for difficulties or inability to perform the task. After initial analysis of the quality of measurements, it became obvious that the majority of the subjects had difficulty with correctly holding the dynamometer due to the relatively heavy weight of the instrument and that the device did not discriminate values below 10 kg. Therefore, after consultations with all the members of the research team, it was decided that the crude HGS values would be omitted in further analysis. Instead, binomial variables were used: YES—the subject was capable of performing the HGS measurement with or without difficulties, and NO—the subject was not capable of performing the HGS measurement. 

### 2.3. Covariates

Sociodemographic characteristics included age, sex, education level, marital status, and place of living. The medical survey included a history of cardiovascular diseases, hypertension, stroke, diabetes, chronic respiratory diseases, osteoarthritis, and osteoporosis. The examination contained elements of the CGA: Katz’s Index of Activities of Daily Living (ADL) [22], Lawton’s Scale for Instrumental Activities of Daily Living (IADL) [23], and the Mini-Mental Scale Examination (MMSE) [24]. The scoring system was the following: ADL (min.–max. score 0–6): dependent (score 0–2), partially dependent (score 3–4), independent (score 5–6); IADL: (min.–max. score 8–24): dependent (score 8–18), partially dependent (score 19–23), independent (score 24); MMSE (min.–max. score 0–30): severe dementia (score < 11); mild or moderate dementia (score 11–23), no dementia (score > 23). Visual and hearing impairments were defined based on the nurse’s evaluation of functional limitations in participants’ everyday life on the condition that glasses or hearing aids were used, if available. 

### 2.4. Statistical Analysis

Statistical analyses were performed using MedCalc Statistical Software v. 14.12.0 (MedCalc Software Ltd., Ostend, Belgium). Statistical significance was set at a *p*-value below 0.05. There has been no data imputation performed. 

Data analyses were performed in the whole group and gender subgroups.

Qualitative data were expressed as percentages. Quantitative data were presented as mean ± standard deviation for the normally distributed variables and median with interquartile range (Q1–Q3) for variables with a skew distribution. Qualitative data were compared with the χ^2^ test, while quantitative data were compared with the *t*-test, U-Mann–Whitney, and ANOVA tests, as appropriate. 

The probability of survival was analyzed with Kaplan–Meier curves to visualize time-to-event (death) trajectories. The analysis of factors potentially affecting the chance for survival was calculated according to Cox proportional hazards regression. In addition to univariate models, several multivariable backward stepwise logistic regression models including age, gender, frailty, and ability to perform HGS measurement were calculated. Hazard ratios (HR) with a 95% confidence interval (±95% CI) and *p*-values were given.

The fact that women outnumbered men, as expected based on the population structure, did not impact the statistical analysis when the whole groups of women and men were compared. It lowered, however, the power of statistical analyses, especially in the analyses of subgroups of males and females. Due to the low proportion of men, separate statistical models for women and men were not developed. Notwithstanding, sex was included in the multivariate analysis of survival, as it had been proven to be related to the chance of survival in the univariate analyses.

## 3. Results

### 3.1. Characteristics of the Cohort

Two hundred eighty-nine (*n* = 289) individuals (77.9% females) were included in the analysis. The mean age of participants was 98.3 years. The sociodemographic characteristics, performance in activities of daily living, mental status, visual and hearing performance, and chronic diseases of the study sample are presented in Table 2. 

The recruited cohort approximately followed population statistics for Poland. According to the population projection for the year 2014, the female to male ratio was the following: at age 94—F:M—3.26:1; at age 97—F:M—3.96:1; at age 100—F:M—4.09:1 [25].

There were no significant differences between men and women in terms of age and hearing impairment. Males were better educated and presented significantly better physical and mental performance than women. Among subjects independent in basic activities of daily living (ADL), only 15% of men and 5% of women were simultaneously independent in instrumental activities of daily living (IADL).

Centenarians constituted 30.3% of the study sample. As expected, the frequency of physical dependency and cognitive impairment increased with age. Every second respondent under 100 years and every fifth centenarian was independent in ADL. The younger group preserved cognitive functions twice more often than the older. There were 24 bedridden subjects in the whole study group (one in eight participants), and 12 among 50 persons living in institutions (one in four subjects). Among centenarians living in institutions, three were bedridden (one in six), and among centenarians living at home, six were bedridden (one in nine).

Based on self-reported morbidity, hypertension, and other cardiovascular diseases were the most frequent health problems regardless of age and sex. The younger cohort was more likely to report osteoporosis and diabetes, while centenarians had a history of stroke more often. Joint and bone diseases were more frequent in women than men.

### 3.2. Frailty and Handgrip Strength Measurement 

One hundred and seventy-two study participants (59.5%) were classified as frail, one-third as prefrail, and less than one in ten as robust. Frailty was more prevalent in women than in men and among centenarians than in the younger cohort (Table 3). Sex-related differences in the distribution of frailty status changed with age but did not reach statistical significance. Among nonagenarians, frailty was diagnosed in 63% of women and 30% of men, and prefrailty in 32% and 48%, respectively. In centenarians, frailty criteria were fulfilled by 75% of women and 50% of men, and prefrailty criteria by 24% and 45%, respectively (in Table 3). One in six younger women and one in four younger men were considered robust, while in the group of centenarians, only one woman and one man remained robust. 

Weakness, the most frequent syndrome of frailty, was reported by 85.4% of the study subjects. Slow walking speed and low level of physical activity characterized seven out of ten participants, while self-reported weight loss characterized only 5.9% of the subjects. Men reported symptoms of frailty less frequently than women, including a low level of physical activity (*p* < 0.001), weakness (*p* < 0.001), and fatigue (*p* < 0.01).

The HGS measurement was performed by 244 study subjects (Table 3). Nine out of ten individuals under 100 years and seven out of ten centenarians could perform the HGS test (*p* < 0.001). The majority of 45 subjects not capable of performing HGS measurement were frail, and only two belonged to the prefrail category. The most frequent causes of failure to measure HGS included the inability to lift and correctly hold the dynamometer due to its weight or hand osteoarthritis.

Table 4 shows the distribution of the characteristics of participants by frailty status. Significant differences were observed in all analyzed variables, except weight loss, which was assumed to be the consequence of a small number of participants reporting weight loss. 

### 3.3. Survivability

**Figure 2:**
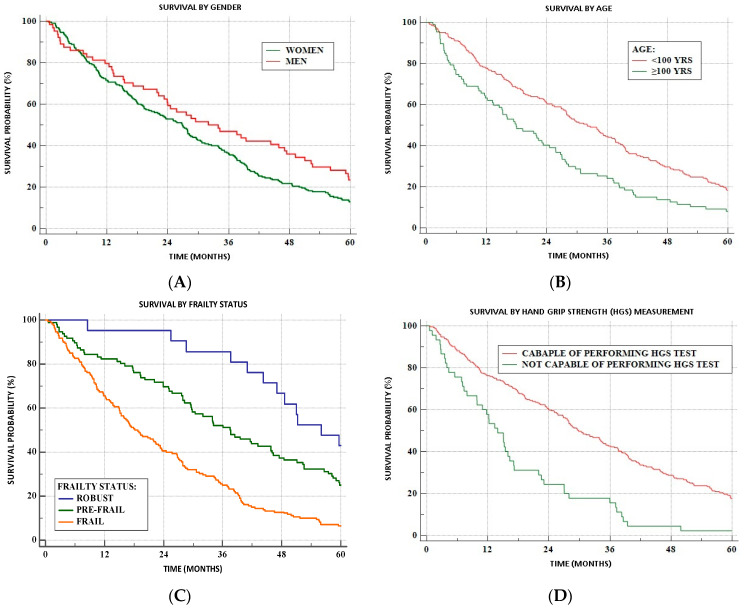
Kaplan–Meier survival curves: (**A**) by sex; (**B**) by age; (**C**) by frailty status; (**D**) by handgrip strength (HGS) test performance.

During the 5-year observation period, 196 women (87.1%) and 49 men (76.6%) have died, including 92.6% of frail women and all the frail men as well as 78.8% of prefrail women and 66.7% of prefrail men. Among robust participants, death was registered in 60% of women and 54.5% of men. Kaplan–Meier survival curves for men and women, younger and older groups, frailty status, and HGS are presented in Figure 2.

In the univariate regression models, the probability of survival was higher for younger individuals, men, and robust as compared to frail subjects as well as participants who were not able to perform the HGS measurement. The difference between prefrail and robust persons did not reach statistical significance. Multivariate Cox proportional backward models incorporated variables that showed a significant correlation with survival in the univariate analysis (Table 5). Models 1, 3, and 5 included the category “age by year”, while models 2, 4, and 6 encompassed the category “age of 100 years and above”. Frailty status was included in models 1, 2, 5, and 6, and the inability to perform HGS measurement in models 3–6. All models comprised sex as a variable. Frailty was the strongest risk factor for mortality in all analyzed models. The inability to perform HGS also correlated with a significantly lower probability of survival. It is worth mentioning that the male sex remained a significant factor only in models in which frailty was not included as a variable (Models 3 and 4). 

## 4. Discussion

### 4.1. Prevalence of Frailty Using Frailty Phenotype

The frailty phenotype defined over two decades ago by Fried et al. [18] remains a valuable tool and is one of the most frequently used clinical scales [15,26]. In the present study, the frequency of frailty symptoms according to the frailty phenotype as well as the relationship between frailty and survivability were established in a relatively large cohort of people in advanced age. The hypothesis that frailty syndrome affected more than half of long-lived individuals was confirmed in Caucasian nonagenarians (55.4%) and centenarians (69%). However, when the sex of the participants was considered, frailty criteria were fulfilled in approximately one in three men and two in three women. The prevalence of frailty was more than two-fold lower in nonagenarian men than women (30% vs. 63%), and sex differences were less apparent in centenarians (50% vs. 75%, respectively). Almost half of all studied men represented prefrailty status, while the state of robustness was very rare in nonagenarian women as well as centenarian men and women.

The prevalence of frailty defined according to the FP was evaluated in older populations with various geographic and cultural backgrounds, including Africa [27], Asia [28,29], Australia [30], Europe [31,32,33], North America [34,35], and South America [36]. Most of the abovementioned studies included participants of a wide age range, namely ≥60 years [27,29,36], ≥65 years [28,30,34], or ≥70 years [31,32], while two studies were dedicated to long-lived individuals: ≥90 years [35] and ≥100 years [33]. Comparability of research on the prevalence of frailty is limited by multiple factors, including methodological approaches, even if the same frailty instrument is used [26]. 

The 5-COOP study compared the prevalence of frailty based on the FP among centenarians in four European countries and Japan [21]. As the methodology of our study followed the procedures of the 5-COOP study, the risk of a methodological bias in the comparative analysis of the results was minimal. In our study, 69% of centenarians fulfilled at least three of five FP criteria and were classified as frail as compared to 51.5% in Sweden, 59.8% in Denmark, 68.2% in Japan, 72.2% in France, and 77.7% in Switzerland [21]. The percentage of robust centenarians varied between 2.8% and 10.8% in the 5-COOP study countries as compared to 2.3% in our study.

The results of our study might also be compared with research dedicated to long-lived individuals in Portugal [33], the USA [35], and China [28]. In the Oporto Centenarian Study, including 50 participants, frailty was diagnosed according to the FP in 60%, more frequently in women [33]. The 90+ Study revealed frailty in 39.5% of US citizens aged 95 and older [35] as compared to 59.5% of all respondents aged 94 and older in our study. The Chinese Longitudinal Healthy Longevity Surveys included significant numbers of nonagenarians and centenarians [28]. In this project, frailty criteria were fulfilled in 39.9% of respondents aged 90–99 yrs and 58.9% of those aged 100 yrs and over, while prefrailty criteria were fulfilled in 49.1% and 36%, respectively. The differences with our study approached 10%, as there were more frail (69%) and fewer prefrail (28.7%) Polish centenarians. Moreover, similarly to our results, the prevalence of frailty was approximately two-fold higher in Chinese women as compared to men, and robust centenarians constituted 5% of the cohort [28]. 

Sex differences in the prevalence of frailty were confirmed also by other authors. The review by Kane et al. [6] showed that a frailty diagnosis based on the FP was more frequent in women than men in 15 out of 16 studies evaluating gender differences. A recent study conducted in Greece reported higher rates of frailty among women as compared to men, irrespective of age [37].

As mentioned in the description of the study sample, the disproportion between women and men in our study (F:M ratio over 3:1) approximately followed the structure of the population of nonagenarians and centenarians in Poland [25]. It did not impact the reliability of statistical analyses but lowered the power of calculations in the comparisons of subgroups of males and females.

### 4.2. Prevalence of Frailty Criteria

Among the five features of the FP, weakness was the most frequently reported by the participants in our study (85.4%). Similar results were found in the 5-COOP study countries: weakness was reported by 77.9–91% of participants [21]. In the group of frail Poles, three symptoms were present in almost all participants: weakness, low level of physical activity, and slow walking speed, as shown in Table 4. In the 5-COOP study, the same three symptoms were accompanied by fatigue in 90% of participants [21]. Among the frail population of Chinese long-lived individuals, low mobility and weakness were also accompanied by exhaustion [28]. The relatively low prevalence of fatigue (equivalent to exhaustion in the FP) in our study (39.5%), indicates that linguistic and cultural factors may play an important role in self-reported questionnaires. The same terms, such as weakness, fatigue, and exhaustion, may have different connotations in various languages [38].

In the present study, weight loss did not correlate with frailty defined as the presence of at least three FP criteria, while the other four criteria showed a strong correlation with frailty status. This result may be explained by the low percentage of participants reporting weight loss but also indicates the importance of objective measurement and monitoring of weight changes, as the subjective perception of older adults might be inadequate.

Repeated evaluation of participants of the English Longitudinal Study on Ageing revealed that slowness was a better predictor of future disability in activities of daily living than weakness or other components of the FP [39]. A face-to-face cross-sectional assessment of the FP in a representative sample of adults aged 60 years and over in Poland showed that slow gait speed was the most frequent symptom (56.3%), followed by weakness (26.9%) [40]. However, when the age group of 90+ was considered, both symptoms were reported at a comparable frequency by the majority of respondents [40].

An interesting approach was presented by Van der Elst et al., who applied a modified Fried phenotype by replacing the measures of slowness and weakness with six questions concerning daily functioning [41]. The modified scale showed considerable concordance with the original FP scale. 

### 4.3. Other Frailty Scales

There is an ongoing discussion on the best set of criteria other than FP to diagnose frailty and differentiate frailty from natural aging-associated physical decline. Popular and widely used instruments for the diagnosis of frailty include the Clinical Frailty Scale (CFS), characterized by seven levels of fitness and frailty [42]; the Frailty Index (FI) based on multiple clinical and laboratory parameters, introduced originally by Rockwood [43] and applied in modified versions [44,45]; and The Survey of Health, Aging, and Retirement in Europe-Frailty Instrument (SHARE-FI), including four questions and HGS measurement [46,47]. 

Recent studies reported interesting results on the comparison of several frailty assessment instruments applied in various countries and different settings. The DO-HEALTH study compared five frailty scales in community-dwelling older adults in five European countries [32]. The prevalence of frailty ranged from 1% to 7% by frailty instrument, and FP criteria defined by Fried et al. were fulfilled by 3% of Europeans aged 70 years and over [32]. 

The FRAILTOOLS project compared eight frailty assessment methods, including the FP, in different outpatient and inpatient settings in five European cities [16,48]. The authors calculated the performance of various frailty scales to predict health-related outcomes, such as worsening in activities of daily living in adults in advanced older age (mean age 83.2 ± 5.4 yrs) in geriatric wards, nursing homes, geriatric clinics, and primary care. Interestingly, none of the instruments showed good sensitivity and specificity in primary care [48]. These findings indicate serious challenges for timely diagnosis of frailty in primary care facing increased shares of longevous patients due to the accelerated aging of the European population. 

Frailty rates changed considerably when several different methods of physical activity assessment were applied to the same group of older adults [49]. Nevertheless, a systematic review by Tolley et al. proved that regardless of the frailty definition, objective measures of physical activity were associated with frailty status [50].

Many authors support the use of multidimensional scales for the assessment of frailty [7,51]. Such an approach is justified if aging is perceived as a progressive accumulation of deficits in multiple dimensions but has limited potential application in routine clinical practice due to the time-consuming nature of the multidimensional assessment. A different approach to frailty links bone health with muscle dysfunction and promotes the term osteosarcopenia as the best descriptor of aging-associated frailty [52]. 

Octogenarians, nonagenarians, and centenarians are special populations of older adults who escape the fate of usual aging and have increased biological reserves, but at the same time are exposed to an extremely high risk of frailty. Therefore, there have been attempts to develop frailty diagnostic instruments dedicated to long-lived individuals, including laboratory biomarkers of biological frailty [53].

It is also worth noting that the concept of frailty is often used as a broad idea, without reference to a specific frailty scale or as a synonym of age-related disability, assessed with commonly used functional scales, including activities of daily living [54].

### 4.4. Correlates of Frailty

Physical frailty is often accompanied by functional decline and sensory impairment as well as cognitive decline. In the present study, frailty correlated with functional performance assessed with ADL and IADL. Significant differences between frail and nonfrail subjects were observed for the total scores; however, when categories of functional performance were used, differences remained significant only in the “independent” categories of ADL and IADL.

Vision impairment was present in 70% of frail subjects, and the same percentage suffered from hearing impairment. Significant differences between frail and nonfrail were seen only in the categories of “no impairment” and “severe impairment”.

In the 5-COOP project, vision and hearing impairments were present in a lower percentage of frail individuals than in our study (52.3% and 43.9%, respectively) [21].

Cognitive performance declined with the age of the participants, and frailty was associated with a high prevalence of cognitive impairment, as only one-third of frail subjects had no dementia according to the MMSE score. The interrelationship of cognitive status and frailty is of clinical importance, as frailty is often accompanied by other mental health problems. A study of centenarians without dementia in Portugal showed that 51.1% of frail individuals presented depressive symptoms, as assessed by the Geriatric Depression Scale, as compared to 21.1% of prefrail and none of the robust centenarians [55]. Our study did not include the assessment of depressive symptoms. Of note, diagnosis of depression in patients with cognitive disorders is extremely challenging and often overlooked in routine medical practice, which may have negative consequences for the clinical course of overlapping syndromes in longevous populations. 

### 4.5. Frailty and Mortality

The concept of frailty is of significant clinical importance, as this geriatric syndrome is associated with multiple adverse outcomes. The second hypothesis of our study concerned the relationship between frailty and mortality. Within 5 years following the geriatric assessment, 92.6% of women and 100% of men classified as frail have died. Frailty was the strongest risk factor for mortality in multivariate regression models, while prefrailty did not reach statistical significance. 

A relationship between frailty and mortality was reported in many recent studies applying the frailty phenotype or its modifications as the measure of frailty [56,57,58,59]. In some of these studies, prefrailty was also a significant risk factor for death [57,59]. 

Interestingly, frailty remained a significant predictor of mortality even if not associated with multimorbidity [59].

The Chinese Longitudinal Healthy Longevity Survey assessed the mortality of 3747 nonagenarians and 3088 centenarians representing the 2002 and 2005 waves of the survey [60]. Frailty that was assessed based on 39 variables significantly increased the relative risk of death. Moreover, it was shown that frailty was a strong predictor of mortality independent of various covariates [61].

Studies of centenarians also confirm the importance of physical performance and frailty status in mortality analysis [62,63]. Survival of long-lived individuals in Poland was evaluated in the Polish Centenarians Program [64]. Longer survival correlated with functional performance in activities of daily living but specific frailty features were not assessed in this project.

Frailty is a dynamic process and does not always follow a unidirectional pattern from robustness via prefrailty to frailty, and transitions of frailty status may influence mortality risk [56]. Frailty is potentially reversible if diagnosed early and favorably at a preliminary stage regarded as prefrailty. In our study, one in three (33.2%) participants were diagnosed as prefrail (29.3% of women, 46.9% of men, 35.2% of nonagenarians, and 28.7% of centenarians). This group could benefit from a frailty prevention program to increase their quality of life and lower the mortality risk.

### 4.6. Handgrip Strength

Loss of muscle mass and strength is the main process underlying the development of frailty [65]. Even though this process is considered a part of physiological aging, there are a lot of factors increasing muscle loss, including intrinsic as well as environmental conditions [66]. The measurement of handgrip strength is a relatively simple, noninvasive, and quick method of assessment of muscle performance, and recent guidelines suggest that the evaluation of muscle strength is the best screening tool for sarcopenia [10]. The loss of muscle power accelerates with age, and population studies indicate that there is a generational shift in reference values of muscle strength [67]. 

The third hypothesis of our study concerned the feasibility of performing reliable HGS measurements in a group of people in advanced older age. The protocol of our research study adopted the methodology of the 5-COOP study in which HGS was skipped from the final analysis due to the low percentage of participants able to perform the measurement [21]. Nevertheless, in our study special attention was paid to the performance of HGS measurements in all participants by a single researcher, who was a trained nurse. Even though most of the participants were able to perform the HGS measurement, the quality of the procedure varied greatly due to technical problems related to holding the dynamometer, and, finally, we decided to omit the crude HGS values in the analysis and apply binomial categories of “capable” and “not capable” of performing HGS as described in the Methods section. Therefore, we failed to prove the possibility of reliable and repeatable HGS measurements in people aged 94 years and older with the mechanical dynamometer.

However, when we included the above binomial categories in the statistical analysis of survivability, the incapacity to perform the HGS measurement proved to be a significant risk factor for mortality in the univariate and multivariate models (Table 5). Published results of studies evaluating the relationship between HGS and mortality indicated an increased risk of death with decreasing HGS values and did not report missing data due to HGS performance issues, but participants in these studies were younger than our study group [68,69]. Technical and procedural issues related to the use of different types of dynamometers, including hydraulic, mechanical, and pneumatic types, were recently described by Lee and Gong [70]. 

### 4.7. Strengths and Limitations of the Study

The strengths of our study include active recruitment procedures to include as many centenarians as possible and careful planning and execution of study procedures. The protocol of the study followed the methodology of the 5-COOP project [21]. The collection of data was performed according to the same procedure for all the subjects, as the research visits were carried out by a single nurse whose activities were dedicated to the project. Data on mortality were obtained from a formal national register. The follow-up of mortality was long (5 years) and concluded before the SARS-CoV2 pandemic.

The limitations of our study include the fact that there was only a single examination of the subjects, which did not allow us to observe frailty trajectories and transitions in the study group. The type of mechanical dynamometer used to examine HGS was not adjusted to the physical capabilities of older adults, but this conclusion was possible only after completing the study. We did not include depressive symptoms prevalence in the analysis. The research protocol did not include any intervention for frail participants. 

### 4.8. Future Perspectives for Frailty Assessment

In the last two decades, frailty has become a hot topic in gerontological research and geriatric care. It is, however, still underestimated in public health and challenging for primary healthcare settings. As none of the eight frailty assessment instruments showed a good performance in primary care in the FRAILTOOL project [48], there is a need for further research and development of simple validated frailty screening tools for different clinical settings. 

Prefrailty screening for younger cohorts of older adults might facilitate rehabilitation and nutritional interventions to enhance healthy aging. Frailty assessment in long-lived individuals might ensure adequate care planning and postponement of disability and might also provide equitable healthcare provision. Moreover, there is a need for research on effective methods of muscle strength measurement in people in advanced older age.

As the integrated approach to care becomes more common than ever, the comprehensive assessment of frailty seems indispensable in clinical as well as societal contexts [71]. Education and training of healthcare professionals to acquire practical skills should be combined with public health strategies based on a frailty-sensitive approach [72]. More research on frailty in various groups of older adults should address differences between unavoidable age-associated frailty and reversible disability as well as support the development of guidelines for clinical practice.

## 5. Conclusions

Frailty assessed with the frailty phenotype was prevalent in nonagenarians and centenarians in Poland and more often affected women than men. Frailty status negatively correlated with functional and cognitive performance and significantly reduced the chance of survival during the five years following the initial evaluation. Handgrip strength assessment with a mechanical dynamometer did not provide reliable measurements, but the inability to perform HGS correlated with the risk of death. Continuing research on frailty is warranted to provide practical guidelines for integrated care and public health.

## Figures and Tables

**Table 1:** Description of frailty criteria according to FP.

Symptom	Criterion	Score
Weight loss	Self-reported weight loss of 5 kg during the past year and/or self-reported weight loss of 3 kg during the past 3 months	0—without weight loss 1—weight loss
Low level of physical activity	Self-reported outdoor activity and/or difficulty walking up a flight of stairs and/or walking a distance of 500 m without rest	0—no difficulty/some difficulty 1—a lot of difficulties or unable to perform at least one walking task
Slow walking speed	Self-reported walking speed	0—fast or normal walking speed 1—slow, very slow, or unable to walk
Weakness	Self-reported difficulty carrying a 5 kg bag	0—no difficulty/some difficulty 1—a lot of difficulties, or unable to perform the task
Fatigue	Self-reported fatigue (when moving or resting)	0—never, rarely, sometimes 1—often, most of the time, or always

Abbreviation: FP—frailty phenotype.

**Table 2:** Characteristics of the study sample: nonagenarians (94–99 years), and centenarians (100 years and over). Subgroups of women and participants aged 94–99 years served as comparators in statistical analyses.

	All (*n* = 289)	Women (*n* = 225)	Men (*n* = 64)	94–99 Years (*n* = 202)	≥100 Years (*n* = 87)
Age [yrs ± SD]	98.3 ± 2.6	98.4 ± 2.5	98.2 ± 2.8	96.9 ± 1.5	101.5 ± 1.5 ***
Living at home [n (%)]	239 (82.7)	180 (80.0)	59 (92.2)	169 (83.7)	70 (80.5)
Living in an institution [n (%)]	50 (17.3)	45 (20.0)	5 (7.8) *	33 (16.3)	17 (19.5)
Education level					
<8 yrs [n (%)]	88 (37.9)	77 (42.5)	11 (21.6)	67 (39.9)	21 (32.8)
8–13 yrs [n (%)]	92 (39.7)	71 (39.2)	21 (41.2)	64 (38.1)	28 (43.8)
>13 yrs [n (%)]	52 (22.4)	33 (18.2)	19 (37.3) **	37 (22.0)	15 (23.4)
Missing data [n]	57	44	13	34	23
ADL score	3.8 ± 1.8	3.6 ± 1.8	4.4 ± 1.8 **	4.1 ± 1.8	3.1 ± 1.8 ***
Dependent [n (%)]	67 (23.2)	55 (24.4)	12 (18.8)	36 (17.8)	31 (35.6)
Partially dependent [n (%)]	102 (35.3)	90 (40.0)	12 (18.8)	65 (32.2)	37 (42.5)
Independent [n (%)]	120 (41.5)	80 (35.6)	40 (62.5) ***	101 (50.0)	19 (21.8) ***
IADL score	13.6 ± 4.9	13.1 ± 4.6	15.2 ± 5.5 **	14.4 ± 5.0	11.6 ± 4.2 ***
Dependent [n (%)]	235 (81.3)	190 (84.4)	45 (70.3)	156 (77.2)	79 (90.8)
Partially dependent [n (%)]	44 (15.2)	31 (13.8)	13 (20.3)	37 (18.3)	7 (8.0)
Independent [n (%)]	10 (3.5)	4 (1.8)	6 (9.4) **	9 (4.5)	1 (1.1) *
MMSE score	22 (15–26)	21 (14–25)	24 (20–26) **	23 (18–26)	17 (11–23) ***
No dementia [n (%)]	119 (42.0)	84 (38.4)	35 (54.7)	100 (49.8)	19 (23.2)
Mild/moderate dementia [n (%)]	126 (44.5)	99 (45.2)	27 (42.2)	82 (40.8)	44 (53.7)
Severe dementia [n (%)]	38 (13.4)	36 (16.4)	2 (3.1) **	19 (9.5)	19 (23.2) ***
Missing data [n]	6	6	0	1	5
Vision impairment					
No impairment [n (%)]	119 (41.5)	82 (36.8)	37 (57.8)	97 (48.5)	22 (25.3)
Mild impairment [n (%)]	50 (17.4)	42 (18.8)	8 (12.5)	34 (17.0)	16 (18.4)
Moderate impairment [n (%)]	73 (25.4)	61 (27.4)	12 (18.8)	49 (24.5)	24 (27.6)
Severe impairment [n (%)]	45 (15.7)	38 (17.0)	7 (10.9) *	20 (10.0)	25 (28.7) ***
Missing data [n]	2	2	0	2	0
Hearing impairment					
No impairment [n (%)]	84 (30.2)	61 (28.5)	23 (35.9)	65 (32.8)	19 (23.8)
Moderate impairment [n (%)]	134 (48.2)	107 (50.0)	27 (42.2)	92 (46.5)	42 (52.5)
Severe impairment [n (%)]	60 (21.6)	46 (21.5)	14 (21.9)	41 (20.7)	19 (23.8)
Missing data [n]	11	11	0	4	7
CVD [n (%)]	185 (64.9)	144 (64.9)	41 (65.1)	130 (65.3)	55 (64.0)
Missing data [n]	4	3	1	3	1
Hypertension [n (%)]	193 (66.8)	154 (68.4)	39 (60.9)	140 (69.3)	53 (60.9)
Past stroke [n (%)]	33 (12.1)	26 (12.2)	7 (11.7)	18 (9.5)	15 (18.1) *
Missing data [n]	16	12	4	12	4
Diabetes [n (%)]	40 (13.9)	30 (13.5)	10 (15.6)	34 (17.0)	6 (6.9) *
Missing data [n]	2	2	0	2	0
Arthritis [n (%)]	77 (26.6)	69 (30.7)	8 (12.5) **	52 (25.7)	25 (28.7)
Osteoporosis [n (%)]	54 (19.9)	45 (21.2)	9 (15.0)	44 (23.0)	10 (12.3) *
Missing data [n]	17	13	4	11	6
COPD [n (%)]	7 (2.4)	5 (2.2)	2 (3.1)	5 (2.5)	2 (2.3)
Missing data [n]	1	1	0	1	0

* *p* < 0.05; ** *p* < 0.01; *** *p* < 0.001. Abbreviations: yrs—years; SD—standard deviation; ADL—activities of daily living; IADL—instrumental ADL; MMSE—Mini-Mental State Examination; CVD—cardiovascular disease; COPD—chronic obstructive pulmonary disease; Q1–Q3—interquartile range.

**Table 3:** Prevalence of frailty, its components, and capability of performing handgrip strength measurement by sex and age. Subgroups of women and participants aged 94–99 years served as comparators in statistical analyses.

	All (*n* = 289)	Women (*n* = 225)	Men (*n* = 64)	94–99 yrs (*n* = 202)	≥100 yrs (*n* = 87)
Frailty status					
Robust [n (%)]	21 (7.3)	10 (4.4)	11 (17.2)	19 (9.4)	2 (2.3)
Prefrail [n (%)]	96 (33.2)	66 (29.3)	30 (46.9)	71 (35.2)	25 (28.7)
Frail [n (%)]	172 (59.5)	149 (66.3)	23 (35.9)	112 (55.4)	60 (69.0)
Frailty criteria					
Weight loss [n (%)]	17 (5.9)	15 (6.7)	2 (3.1)	13 (6.4)	4 (4.6)
Low physical activity [n (%)]	200 (69.2)	172 (76.4)	28 (43.8) ***	133 (65.8)	67 (77.0)
Slow walking speed [n (%)]	204 (70.6)	162 (72.0)	42 (65.6)	137 (67.8)	67 (77.0)
Fatigue [n (%)]	68 (23.5)	62 (27.6)	6 (9.4) **	43 (21.3)	25 (28.7)
Weakness [n (%)]	246 (85.4)	203 (90.6)	43 (67.2) ***	166 (82.2)	80 (93.0) *
Handgrip strength measurement					
Performed [n (%)]	244 (84.4)	186 (82.7)	58 (90.6)	182 (90.1)	62 (71.3) ***
Not performed [n (%)]	45 (15.6)	39 (17.3)	6 (9.4)	20 (9.9)	25 (28.7) ***

* *p* < 0.05; ** *p* < 0.01; *** *p* < 0.001.

**Table 4:** Characteristics of the study sample by frailty criteria. The robust subgroup served as the comparator in statistical analyses.

	Robust (*n* = 21)	Prefrail (*n* = 96)	Frail (*n* = 172)	Comparison of Subgroups
Age [yrs ± SD]	96.3 ± 1.7	98.0 ± 2.1 **	98.7 ± 2.8 ***	*p* < 0.001
Min–max	94–100	94–102	94–106	
Female [n (%)]	10 (47.6)	66 (68.8)	149 (86.6)	*p* < 0.001
Male [n (%)]	11 (52.4)	30 (31.3)	23 (13.4) ***	
ADL	5.8 ± 0.4	4.8 ± 1.3 ***	3.0 ± 1.8 ***	*p* < 0.001
Dependent [n (%)]	0	4 (4.2)	63 (36.6)	*p* < 0.001
Partially dependent [n (%)]	0	31 (32.3)	71 (41.3)	
Independent [n (%)]	21 (100)	61 (63.5) **	38 (22.1) ***	
IADL	19.5 ± 3.9	16.2 ± 5.1 **	11.4 ± 3.4 ***	*p* < 0.001
Dependent [n (%)]	9 (42.9)	62 (64.6)	164 (95.3)	*p* < 0.001
Partially dependent [n (%)]	8 (38.1)	28 (29.2)	8 (4.7)	
Independent [n (%)]	4 (19.0)	6 (6.2)	0 ***	
MMSE [score (Q1–Q3)]	23 (21–26)	24 (20–27)	20 (14–25) **	*p* < 0.001
No dementia [n (%)]	10 (47.6)	53 (55.2)	56 (33.7)	*p* < 0.001
Mild or moderate dementia [n (%)]	11 (52.4)	39 (40.6)	76 (45.8)	
Severe dementia [n (%)]	0	4 (4.2)	34 (20.5) *	
Missing data [n]	0	0	6	
Vision impairment				
No impairment [n (%)]	17 (81.0)	51 (53.7)	51 (29.8) ***	*p* < 0.001
Mild impairment [n (%)]	1 (4.7)	20 (21.0)	29 (17.0)	
Moderate impairment [n (%)]	3 (14.3)	19 (20.0)	51 (29.8)	
Severe impairment [n (%)]	0	5 (5.3)	40 (23.4) ***	
Missing data [n]	0	1	1	
Hearing impairment				
No impairment [n (%)]	6 (28.6)	42 (44.7)	36 (22.1) ***	*p* < 0.001
Moderate impairment [n (%)]	13 (61.9)	40 (42.5)	81 (49.7)	
Severe impairment [n (%)]	2 (9.5)	12 (12.8)	46 (28.2) **	
Missing data [n]	0	2	9	
Frailty criteria				
Weight loss [n (%)]	0	0	17 (9.9)	*p* = 0.07
Low level of physical activity [n (%)]	0	31 (32.3)	169 (98.3)	*p* < 0.001
Slow walking speed [n (%)]	0	45 (46.9)	159 (92.4)	*p* < 0.001
Fatigue [n (%)]	0	0	68 (39.5)	*p* < 0.001
Weakness [n (%)]	0	78 (81.3)	168 (98.2)	*p* < 0.001
Handgrip strength measurement				
Performed [n (%)]	21 (100)	94 (97.9)	129 (75.0)	*p* < 0.001
Not performed [n (%)]	0	2 (2.1)	43 (26.0)	

* *p* < 0.05; ** *p* < 0.01; *** *p* < 0.001 vs. robust. Abbreviations: yrs—years; SD—standard deviation; min—minimal; max—maximal; ADL—activities of daily living; IADL—instrumental ADL; MMSE—Mini-Mental State Examination; Q1–Q3—interquartile range.

**Table 5:** Results of univariable and multivariable Cox proportional hazards regression models (backward models) explaining survival.

Variable	Univariable Model HR (95% CI)	Multivariable Model 1 HR (95% CI)	Multivariable Model 2 HR (95% CI)	Multivariable Model 3 HR (95% CI)	Multivariable Model 4 HR (95% CI)	Multivariable Model 5 HR (95% CI)	Multivariable Model 6 HR (95% CI)
Age by year	0.889 (0.847–0.932) ***	0.902 (0.861–0.946) ***	Not included	0.915 (0.869–0.963) ***	Not included	0.919 (0.874–0.966) ***	Not included
Age ≥ 100 yrs	0.617 (0.476–0.799) ***	Not included	0.647 (0.499–0.839) **	Not included	0.752 (0.567–0.997) *	Not included	0.740 (0.558–0.982) *
Men	1.393 (1.038–1.869) *	ns	ns	1.356 (1.009–1.821) *	1.368 (1.019–1.837) *	ns	ns
Prefrail	0.609 (0.366–1.014) ^	ns	ns	Not included	Not included	ns	ns
Frail	0.328 (0.200–0.539) ***	0.474 (0.368–0.611) ***	0.466 (0.362–0.599) ***	Not included	Not included	0.503 (0.388–0.653) ***	0.498 (0.384–0.645) ***
Individuals not capable of performing HGS measurement	0.338 (0.237–0.481) ***	Not included	Not included	0.507 (0.357–0.721) ***	0.472 (0.329–0.676) ***	0.631 (0.442–0.902) *	0.599 (0.415–0.865) **

^ *p* < 0.10; * *p* < 0.05; ** *p* < 0.01; *** *p* < 0.001; ns—not significant; yrs—years.

## Data Availability

The data presented in this study are available on request from the author mpuzianowska@imdik.pan.pl (M.P.-K.).
